# American Medical Association

**Published:** 1855-06

**Authors:** 


### EDITORIAL.

*American Medical Association:*

FIRST DAY.

This Association commenced its Eighth Annual Session May 1st, at 11 A. M., in the Musical Fund Hall. The spacious saloon was divided into two parts by a railing; one apartment being exclusively appropriated to the delegates, and the other part to spectators; the reporters of the press being amply provided for.

We observed in the reception room in the basement story, a block of marble, designed for the Washington National Monument.—The block is finished in a chaste and beautiful style, and bears the following inscription:

"AMERICAN MEDICAL ASSOCIATION.
INSTITUTED MDCCCXLVII.
VINCIT AMOR PATRIAE.

On the face of the stone, cut in alto-relievo, is a well executed representation of Hypocrates, the father of Medicine, refusing a bribe tendered him by Artexerxes King of Persia. There are no less than eleven figures in the group. The stone is four feet long, two feet thick and one foot eight inchos high, and the workmanship a handsome specimen of American sculpture. The work was performed by J. A. Beck, a young man, who has since proceeded to Rome, to cultivate his talent as a sculptor.

At half-past eleven o'clock, the Association was called to order by the President, Dr. CHARLES A POPE, of St. Louis, who, on taking the Chair, invited the Ex-President and Ex-Vice Presidents of the Association to take seats on the platform.

Dr. Francis West, of Philadelphia, and Dr. E. S. Lemoine, of St. Louis, officiated as Secretaries.

Dr. Isaac Hays, from the Committee of Arrangements, stated that on behalf of the Medical profession of Philadelphia, he gave a warm and cordial reception to the delegates assembled. The Committee have done their best to make the delegates as comfortable as possible, during their sojourn in the city. To do so, is not only a gratification, but a duty. Eight years have elapsed since the organization of this Association, and we well know, that our delegates have been received in different parts of our country during that period of time, with generous hospitality. We beg leave to assure the delegates in attendance, that it is our most earnest desire to do everything in our power to return the compliment to the assembled wisdom of the Medical profession.

On motion of Dr. Hays, the roll was called by Dr. F. West.—The following is a full list of the delegates :—

MAINE.

Maine Medical Association—A. J. Fuller, G. S. Palmer, Alpheus F. Page, N. P. Monroe, Jas. C. Weston, Nathan Emerson.

Penobscot Medical Society—Horatio N. Page, J. P. Dickinson.

NEW HAMPSHIRE.

New Hampshire Medical Society—Silas Cummings, Thos H. Marshall.

Strafford District Medical Society—David T. Parker.

VERMONT.

Vermont Medical College—George T. Elliott.

Grafton District Medical Society—Israel Hinchley.

MASSACHUSETTS.

Massachusetts Medical Society—Henry Willard, W. M. Cornell, E. P. Eastman, Alex. Thompson, Geo. Hayward, Winslow Lewis, Jeremy Stimson, Martin Root, Anson Hooker, Jonathan W. Bennis. Horace Richardson, Ephraim Buck, Benj. E. Cotton, C. P. Fiske, W. H. Page, Edward B. Moore, Jasper H. York, A. Le Baron Monroe, Gilman Kimball, S. G. Burnap, John Geo. Metcalf, Robert T. Davis, Augustine Shurtleff, James Farnum, Benj. Cox, Jr., John Homans.

Middlesex County District Medical Society—Anson P. Hooker.

Thurber Medical Association—Ambrose Eames, Allen C. Fay.

Boston Medical Association—E. D. G. Palmer, S. L. Sprague.

Boston Society for Medical Observation—Calvin Ellis, J. N. Borland.

Medical Department Harvard University—D. H. Storer, Ed. H. Clarke.

Permanent Member—Jno. Green.

RHODE ISLAND.

Permanent Member—Usher Parson, Providence.

Rhode Island Medical Society—Joseph Mauran, Ariel Ballou, Jas. H. Eldridge.

CONNECTICUT.

New Haven county Medical Society P. Jewett, Geo. C. Buddington, A. B. Ives, H. W. E. Matthews, Reynolds Webb, Leonard J. Sanford, John Nicoll.

Litchfield county Medical Society—J. G. Beckwith, Samuel T. Salisbury, James Welsh.

Fairfield county Medical Society—S. S. Noyes.

Medical Institute, Yale College—Chas. Hooker, Jonathan Knight.

Connecticut State Hospital—E. H. Bishop.

New London county Medical Society—Ashbel Woodward, John D. Ford

Windham county Medical Society—Lewis Williams.

Tolland county Medical Society—Francis L. Dickinson, Alden Skinner. Permanent Member—Reynolds Webb.

NEW YORK.

Medical Society of Erie Co.—Jas. M. Newman.

New York County Medical Society.—Isaac Wood, D. Meridith Reese, Alfred C. Post.

Herkimer County Medical Society.—Wm. H. H. Parkurst, A. F. Dolittle.

Binghamton Academy of Medicine.—George S. Little.

Madison County Medical Society.—A. D. Saunders.

Academy of Medicine.—F. Campbell Stewart, David Greene, Jno W. Corson, W. Rockwell, Jr., Foster Jenkins.

Medical Association of Southern and Central New York—Jno. Gay Orten, R. O. Crandall.

New York Medical College.—B. Fardyce Barker, Horace Green.

Buffalo Medical Association—Charles H. Wilcox, Frank H. Hamilton.

New York State Emigrant Hospital—Thomas Addis Emmet, Ernest Schilling.

Society of German Physicians—Ernest Krackowizer, Theo. A. Tellkampt.

Onedia county Medical Society—Jno. McCall, N. A. Dering.

Monroe county Medical Society—Jno. Reed.

New York Hospital—John Watson. Joseph M Smith.

Richmond county Medical Society—W. C. Anderson.

New York State Medical Society—E. W. Armstrong.

Broom county Medical Society—Thomas Jackson.

New York Pathological Institute—T C. Fennell.

Permanent members—Caleb Budlong. Henry S. West, E. H. Davis A. Baker, Jr., H. M. Eastman.

PENNSYLVANIA.

Philadelphia College of Physicians—Isaac Hays, G. B. Wood, R. A. Given, Ed. Hartshorne. J. B. Biddle, Francis West, Franklin Bache. Jno. Neill, Fras. G. Smith, Jr., Jas. Carson, Paul Beck Goddard, Jos. Carson, J. Rodman Paul.

Pennsylvania State Medical Society—Charles Innes, Henry Carpenter, G. Emerson, David O. Moser, Jno. A. Martin, Robert K. Smith.

University of Pennsylvania—Hugh L. Hodge, Samuel Jackson.

Jefferson Medical College—Joseph Pancoast, Charles D. Meigs.

Pennsylvania Medical College—David Gilbert. J. M. Allen.

Philadelphia College of Medicine—Isaac A. Pennypacker, Henry Hartshorne.

Pennsylvania Hospital—George W. Norris

Wills' Hospital—S. Littell.

St Joseph's Hospital—Alfred Stille.

Episcopal Hospital—John J. Reese.

Philadelphia Hospital, Blockley—J. L. Ludlow, Henry H. Smith.

Philadelphia county Medical Society—Robert P. Thomas, Samuel L. Hollingsworth, William V. Keating, Caspar Wistar, Benjamin S. Janney, Thomas H. Yardley, D. Francis Condie, D. Paul Lajus, Wilson Jewell, John Bell, Thomas F. Betton, Sam'l Jackson, William Byrd Page, Samuel Lewis, Anthony E. Stocker, Wm. H. Klapp, Ellwood Wilson, John Corney, James V. Emlon, Arnold Naudain.

Northern Medical Association—Levi Curtis, William Maybury, R. H. Townsend, N. L. Hatfield, J Henry Swaltz.

Western Clinical Infirmary—Thos. Howsen Bache.

Philadelphia Dispensary—James M. Corse.

Chester county Medical Society—M. Emanuel, C. J. Morton.

Lancaster city and county Medical Society—J. B. Stubbs, P. Cassidy, J. K. Rand, Thomas Ellmaker, Robert Duncan.

Lancaster county Hospital—John L. Atlee.

Huntingdon county Medical Society—J. M. Gemmell.

Medical Society of city of Reading and county of Berks—Frank M. Heister, P. G. Bertolet, William Gies, Edward Wallace.

Bucks County Medical Society—John Dyer.

Moyamensing House of Industry—Jacob Da Costa.

Lehigh Medical County Society—Charles H. Martin.

Montgomery County Medical Association—Hiram Corson, C. Shoemaker, John L Foulke.

Blair county medical society—Wm. R. Finlay.

Mifflin county medical society—Abram Rothnock. Permanent members—Henry Bond, Geo. J. Ziegler—Rene La Roche, B. Horner Coates, Winthrop Sargent.

Parisian medical society—Walter F. Atlee.

NEW JERSEY.

Camden county medical society—A. D. Woodruff.

Camden city medical society Thos. F. Cullen.

Cumberland county medical society—Isaac H Hampton.

New Jersey state medical association—R. M. Cooper, Lewis Condict.

Burlington county medical society—Henry H. Longstroth, Geo. Godell, J. P Coleman.

Hudson county medical society—Charles Cook, John Thompson.

Sussex county district medical society—A. D Marford, John R. Stuart.

Gloucester county medical society John R. Sickler.

Permanent members—G. H Taylor, Wm. Elmer, S. W. Butler.

DELAWARE.

Delaware state medical society—Robert H. Clarke, Henry F. Asker, John Merritt, Gove Saulsbury.

New Castle county medical society—Jas. Couper, J. W. Thompson.

Willmington medical association—Wm. R. Bullock, James F. Wilson.

Permanent member—L. P. Bush.

MARYLAND.

Baltimore Infirmary—Richard H. Thomas.

Medical and chirurgical faculty of Maryland—Peregrine Wroth Joel Hopkins, Samuel P. Smith, A. M. White, Washington Duvall, Chas Macgill.

Medical and surgical society of Baltimore—A Snowden Piggott, Wm. H Baltzell.

St. Mary's county medical association—Thomas Matthews.

University of Maryland—George M. Miltenberger.

Talbot county medical society—J. E. M. Chamberlain, Thomas W. Martin, Edw. M. Hardcastle.

DISTRICT OF COLUMBIA.

National medical college—Lewis H. Steiner, Thomas Miller.

Washington city Infirmary and Hospital—B. Johnson Hellen.

Medical Society of District of Columbia—Grafton Tyler, Alex. J. Simmis, Jno. C. Riley, Harvey Lindsley, Jas. C. Hall, C. Bayle, G. M. Dove.

VIRGINIA.

State medical society of Va.—Perterfield Trent, J. Lewis Dorset.

Medical college of Va.—Charles Bell Gibson, B. R. Wellford.

Winchester medical college—Alfred B. Tucker, Hugh McGuire.

Prince Edward county medical society—James Lyle.

Medical Department University of Virginia—J. L. Cabell, B. W. Allen.

Petersburg medical faculty—James W. Smith, J. F. Feebles, Pitman C. Spencer.

SOUTH CAROLINA.

South Carolina Medical Association—T. T. Robertson, J. F. M. Geddings, N. H. Gibbes, W. W. Mobley, R. F. Michel, John L. Dawson, R. S. Bailey, C. Happoldt.

Medical College of the state of South Carolina—Thomas G. Prioleau, Henry R. Frost.

Columbia Medical Society—Samuel Fair.

Charleston Medical Institute—F. M. Robertson.

GEORGIA.

Savannah Medical College— Richard D. Arnold.

ALABAMA.

Alabama State Medical Association---William M. Bolling, P. H. Cabell.

TENNESSEE.

Permanent Member---Frank A. Ramsay.

Tennessee state Medical society—R. C. Foster.

University of Nashville---J. Berrien Lindsley.

KENTUCKY.

Covington medical society---C. J. Blackburne.

Louisville marine hospital---J. C. Johnston.

University of Louisville---T. G. Richardson.

Kentucky state medical society---David Thompson, J. T. Bradford.

Kentucky school of medicine---Joshua B. Flint, A. B. Cook.

OHIO.

Ohio state medical society---Levi D. Scheetz, Isaac C. Williams, R. Hills, R. B. Leonard, J. F. Potter, Ephraim Gaston R. R. McMeens.

Belmont county medical society---Ephraim Gaston, J. D. Cotton, Abel Cary, A. Dunlap.

Miami medical college---R. D. Mussey.

Erie county medical association---Daniel Tilden.

Clinton county medical society---T. W. McArthur.

Medico-Chirurgical seciety of Cincinnati---Alexander M. Johnston, Wm. Clendennin.

INDIANA.

Union medical society, Northern Indiana---M. M. Latta.

Indiana state medical society---Joel Pennington, Thos. W. Florer, E. Murphy.

Kosciusko county medical society---William E Sarber.

ILLINOIS.

Rush medical society---N. S. Davis.

Cook county medical society---J. W. Freer.

Lasalle county medical society---Joseph Stout.

Illinois state medical society---Daniel Brainard, A. H. Luce, S. W. Noble, Rudolphus Rouse.

MISSOURI.

St. Louis medical association---Chas. A Pope.

St. Louis medical society---L. P. Perry, T. E. Massey, E. S. Lemoine.

IOWA.

Medical department Iowa University---J. E. Sandborn.

WISCONSIN.

Alfred L. Castleman, J. J. Brown.

Milwaukee medical association---J. B. Dousman.

MICHIGAN.

N: B. Stebbins, H. Taylor, J. H. Beech.

Detroit medical society---Wm. Brodie, Zina Pitcher.

University of Michigan---A. B. Palmer.

UNITED STATES NAVY.

T. Dillard, Jas. M. Greene, Samuel Barrington.

---

On motion, the President of the Association was invited to deliver an address. He spoke as follows :

Gentlemen :—With feelings of grateful pleasure, I meet you, and greet you, on this occasion.

For high and useful purposes have we assembled from the wide extent of our beloved country. The elevation of a noble profession—the promotion of science—the good of humanity—these have been, are, and will continue to be, the objects of our Association. Whether we have, thus far, done much or little, our sole aim has been the advancement of the best interests of ourfellow-men. I shall not assert that we have done as much as we might have done or that the course hitherto pursued by us, is so perfect, as to admit of no improvement. Were such the fact, and were the Association a firmly established institution, I might have experienced more hesitatton in the selection of a theme for the present occasion. And since we cannot, as yet, I think, urge such a claim, the few suggestions which I shall offer, are made with becoming diffidence, but at the same time with a deep sense of their importance to the welfare and perpetuity of our Association.

Some strictures on our proceedings, in Medical and other journals, have appeared within the last year, as well as in previous years. I shall not here blame the authors of them. They are, doubtless, as proud of our noble profession as we, and equally with us, anxious for the advancement of its interests and its honor. I thank them for their suggestions. All of us are ready to hear them and to profit by them. If any more actual mode of arriving at truth can be devised, than that which we have heretofore pursued, all of us are ready to follow it, and would rather thank than quarrel with those who may propose it.

Physicians have an almost superhuman mission to fulfil. The goal of their ambition, and their hopes, and their duty, stands at the *ultima thule* of human capacity—nay rather beyond it. It cannot, indeed, be said that their duties are beyond their powers, but their ambition, their hopes, their wishes, certainly are. They would gladly know, not only all the secrets of organization, but those also of Physiology, Pathology and Therapeutics. To arrive at such knowledge is, perhaps, beyond the attainment of the human mind. Multiform are the elements which enter into the problem of health and disease. Health is, itself, a constant change of composition—diseases are ever-varying changes, supervening on this.

Do we not know, with all our advancement, and after all the toil of our predecessors for two thousand years, the exact changes n which any disease, the fevers for instance, consists? And even when we shall have learned these, so as to understand them as well as the most ordinary chemical changes, the ever varying character of most diseases, and the inward distributing influences upon them of the mental and moral emotions, would require to follow them, a continued stretch and power of intellect, which it is doubtful if man be capable. This exactness of knowledge is not, I grant, necessary to the very successful practice of medicine. Our profession can render great and important services to man without it, but with it, it would be still more serviceable. To it our ambition tends. To this perfect knowledge we aspire. Athough we may never reach, we can yet eternally approach it. In the vast region of our researches, there is no probability that human genius will ever, Alexander like, weep for the want of unconquered provinces. Beyond the conquests of the future heroes of the profession, there will always be a boundless field for the ambitious and philanthropic explorer. In the language of a western student, "the science of Medicine, like the liver of Prometheus, is sufficient to glut the eagles of all time."

The object of this Association is to do something to advance the profession towards the far-distant goal of perfection—to aid the solution of some of the problems and enigmas of life and organization—to add some material to the growing temple, whose foundations were so firmly laid by the Coan sage—and to do its part, as best it may, in the cause of humanity. Nor do I think that so far, it has altogether failed. Many valuable contributions to science have been elicited—professional ambition has been stimulated—an *esprit-de-corps* has been successfully evoked and established. The strength of the profession has acquired additional power by the union of its members. This association has been to physicians, what the railroad and electric wires are to commerce, and the interchange of useful knowledge to states and nations. It has made us one, and, as I have just remarked, in unity there is power. This association has stimulated thought. Chaotic and void would forever remain the masses of facts, accumulated by the observations of ages, but for the co-ordinating and logical power of reason. It sits in judgment on the silent phenomena, as a "refin r of fire, and a purifier of silver." It forces the voiceless facts to mount the tripod of the oracle, and speak forth words of wisdom. The scalpel, the crucible, the microscope, may be subsidiary to its purpurposes and ends, but they cannot supply its place. Fixed and patient thought, in medicine, as in other departments of science, is the Aladdin's lamp that lights the footsteps of the discoverer. To stimulate attention and thought, is to accelerate many a new discovery—to hasten the advent and establishment of important principles yet in the womb of the future. May not our Association do this more effectually than it has hitherto done?

Let all the contributions be read and attentively considered. Such a course would certainly be more encouraging, as well as more respectful, to their authors. Let the reports be deliberately and fully discussed, and let them go forth to the world with the sanction or the criticisms of the Association. This would require time, it is true, but if we have time to meet at all, surely a few days would make but little difference. The good that would be effected would yield a ten-fold compensation for the time employed. Every one must admit that three or four days is too short a time for the Association rightly to fulfil its annual mission.

I would, moreover, respectfully suggest that time be taken for the discussion of some of the leading topics of medical philosophy. Amongst these, may be mentioned the nature, causes and treatment of cholera, yellow fever, *et cetera*—Hygiene, and the laws of health affecting masses of men—Quarantine—the causes of mortality among children - the chemical and vital doctrines of life. Questions like these, indicated a year in advance for discussion, would excite a carefulness of investigation, and a degree of attention and thought which could not fail to clear away much of the darkness and doubt in which they are yet shrouded. Nothing so sharpens the intellectual powers as public debate. It fixes attention, and strains to the utmost every faculty. I have no hesitation in saying that facts enough have been accumulated to establish great and general principles, of which the medical world is yet in ignorance or doubt. Nothing would contribute more to demonstrate these principles than the collision of natural intellects in public debate. What a mass of facts, and argument, and demonstration would be brought to bear on any of the subjects alluded to, if some of the best minds in the profession were to debate them, after a year's preparation! Observed facts are the crude materials of science—the intellect is the master builder of its august temple.

I make these suggestions for your consideration. All the scientific meetings in this country and in Europe, employ more time than ours has hitherto employed. Eventually we must protract our sessions, if we would render them serviceable to science as they may be. No member of the association will be required to remain longer than suits his wishes or convenience. Some fifty or sixty, more or less, would always be found to listen with eagerness to scientific papers, and engage with pleasure in scientific discussion.

The time has probably arrived, for a change in our plan of organization, which will admit of the selection of a permanent place for the future meetings of the Association. There are evident advantages incident to both the migratory and stationary plans. These might, perhaps, be easily reconciled and secured. A proposition, if I mistake not, was made some years ago, by the Smithsonian Institution, and I would respectfully suggest, whether it would not be in accordance with the best interests of the Association, to hold biennial meetings in Washington, and the alternate one, as now, at different points of our common country. We might thus secure all the advantages of a fixed abode, in the way of preserving the archives, making collections, etc., whilst by meeting in different localities, we could not fail to excite that wide-spread interest among the profession, and obtain such accessions of new members as would greatly enhance the high and useful objects of our Association. Should this proposal meet with your approbation, I would further intimate that policy would perhaps require the meetings of the Association at the National Capital to be held in the years of the short sessions of Congress.

I shall say but little of the legislative duties of the Association. I shall say nothing of the propriety or impropriety of getting laws passed to regulate the practice of medicine, and furnish standards for candidates for the Doctorate. Perhaps the Association can do but little in this respect. Ours is a popular government, and the people are disposed to allow the largest freedom in relation to everything pertaining to medicine, medical schools, and physicians. Laws passed against quackery one year are revoked the next. Our country is the paradise of quacks. All good things have their attendant evils, and this unbridled liberty is one of the evils of a popular government. May we not hope however that even this evil may disappear, as general education and the cultivation of the masses advance? At any rate the people are not yet disposed to put down the quacks, nor to require too high a degree of qualification for those of the regular profession. After all, laws can make only mediocre physicians. They can require the candidates to know only so much—to be qualified to a certain degree; and this degree will always be far lower than that to which the true lovers of knowledge would attain, without any legislation on the subject. The greater lights of the profession cannot be manufactured after any system of legislative enactment. Thirst of knowledge, self-love, philanthropy, burning ambition---these make the great physician and surgeon. These have made all the worthies of the past---not legislation. Legislation cannot drive the drone to the proud heights of professional eminence. When these heights are reached, it will be seen that the successful aspirant has been stimulated by a stronger power.

To him the laurel blossoms of renown and the life-giving missions of his art are dearer and more attractive than was the mystic bough of the sibyl to the eager Æneasor; than the golden apples guarded by sleepless dragons, to the Hesperean daughters.

Whatever course you may think proper to pursue, I am sure that your objects will be, the advancement of science, the good of mankind, the honor and glory of the profession. We have the dignity and character of a noble calling to sustain—of a profession which has numbered, for two thousand years and more, some of the wisest and best men in all countries and all times. It is no trivial matter, to sustain the rank and respectability of a vocation which can boast of a Hippocrates---a Harvey---a Huntor---of the most erudite and beneficent sages and philanthropists the world ever saw---of a profession which has furnished to every nation its *clarem et venerabile nomen.*

On the eve of the battle of the pyramids, Napoleon exclaimed,

Soldiers ! from the height of yon monuments forty centuries look down upon you. Gentlemen, from the heights of past ages, countless worthies of onr God-like profession point and beckon to a goal more elevated than that which atracts legislators and conquerors, Solons and Cesars.

---

Dr. N. S. Davis, Vice-president, was called to the chair.

On motion of Dr. J. B Biddle, a vote of thanks was passed for the able and eloquent address of the President.

Dr. Hays, from the Committee of Arrangements, recommended that the time be fixed for the sessions of the Association, from 9 o'clock A.M., to 8 P.M.. with one hour recess. The report was adopted.

Dr. Hays also reported that the delegates are invited to visit the Pennsylvania Hospital for the Insane, and that omnibuses would be ready at a quarter past 4 o'clock precisely, in front of the Musical Fund Hall, the Girard House, and the La Pierre House.

On motion, a recess of five minutes was taken, for the purpose of nominating a committee on Permanent Organization.

At this period the scene became quite animated, the delegates from the several States assembling in different parts of the room, to make their respective nominations.

VISITATIONS.

At noon of this day, the delegates will visit Independence Hall where they will be received by the Hon. R. T. Conrad. At 4 o'clock this afternoon, they will visit Fairmont and the Girard College. To-morrow afternoon, the Philadelphia Hospital at Bleckley.

Friday the Asylum for the blind.

During the session, the delegates will also visit the University of Pennsylvania, and other medical institutions, notable places, &c.

Dr. Francis Condie moved that all permanent members who did not pay their annual dues of membership in the proper time, in consequence of a want of notification, but who have since paid, be re-admitted as permanent members of the association.

After a brief discussion, Dr. Watson moved an amendment, that no permanent member of this association can be deprived of his privileges on account of failing to contribute any funds at any meeting at which he is not in attendance.

Dr. White moved to refer the whole subject to a committee of three, to report, &c. Adopted.

The President appointed Dr. White of Buffalo, Dr. Watson of New York and Dr. D. F. Condie of Philadelphia.

The delegates from the several states reported the following names as the committee on nominations.

Maine, A. J. Fuller; New Hampshire, Silas Cummings; Vermont, Israel Hinckley; Massachusetts, C. P. Fiske; Rhode Island, Joseph Mauran; Connecticut, P. A. Jewett; New York, John McCall; Pennsylvania, J. B. Biddle; New Jersey, Lewis Condict; Delaware, James W. Thompson; Maryland, Chas. McGill; District of Columbia, Thomas Miller; Virginia, B. R. Willford; North Carolina, O. F. Manson; South Carolina, P. C. Gaillard; Georgia, Richard D. Arnold; Alabama, P. H. Cabell; Tennessee, J. Berrien Lindsley; Kentucky, C. J. Blackburne; Ohio, R. S. Hills; Indiana, Joel Pennington; Illinois, J. V. Z. Blaney; Michigan, A. B Palmer; Mississippi, L. P. Perry; Iowa, J. E. Sanborn; Wisconsin, J. B. Dousman.

Dr. Stewart, of New York, offered a resolution providing that the nominating committee be instructed to present the names of three members for the office of President, to be elected by ballot, the lowest candidate to be dropped.

Dr. Arnold moved to amend by adding the words, providing that no one has a majority.

Dr. Stewart accepted the amendment.

After a brief discussion the subject was laid on the table.

A motion was now made that the nomination committee be requested to meet down stairs in the refresment room. Adopted.

It was moved and adopted that the committee be instructed to recommend a place of meeting for the next annual convention.

Dr. Pitcher, of Detroit, presented a communication from the faculty of that place, asking that the next meeting of the association be held there. Referred to committee on nomination.

Invitations to meet at Chicago and Nashville, and so much of the address of the President as relates to the places of next meeting, were also referred to the nominating committee.

Dr. D. D Thompson, of Kentucky, moved that the regular order of business be suspended for the purpose of taking into consideration a proposed amendment to the constitution, offered at the last meeting of the association. The motion was lost.

The association now proceeded to consider the nominations of members by invitation, and a motion to refer the subject to the committee of arrangements prevailed.

The chair announced the next business in order, the reading and consideration of the stated annual reports from the standing committees.

Invitations to visit the United States Mint, and the Academy of Natural Sciences, were read and accepted.

Dr. Rouse, of Illinois, moved that the thanks of the association be extended to the gentlemen who have extended the invitations to visit the several places above named. Adopted.

Dr. R. La Rouche, from the committe on prize essays, submitted the following report:

The committee on prize essays and volunteer communications report:

That the essays received by them were six in number. These the committee have submitted to a patient and careful examination, and with pleasure acknowledge the greater number are written with ability, and evince a commendable degree of familiarity with the subject under consideration. But while freely admitting thus much, and expressing the opinion, that the essays in question if placed in a printed form before the public, would do great credit to their respective authors, the committee have, after mature deliberation, concluded that only one among the number to which their attention was called, presented those qualities which in their opinion should entitle essays to the award of the prizes offered by the association.

The committee have in consequence decided on awarding but one prize. The essay selected is entitled "Statistics of Placenta Prævia." It is one of considerable merit. It evinces an unusual degree of industry in the collection and an uncommon talent in the arrangement and classification of facts from which the author draws practical deductions of high value. The essay is accompanied with extensive tables of cases which ensure its completeness and enhance in no small degree its usefulness.

The essay bears the following motto, "Homines Mulla re proprius ad Deos accedunt quam hominibus salutem dando."

The name of the author was now announced to be James T. Trask, M. D., White Plains Westchester county, of New York. The announcement was received with applause.

Dr. Reyburn, of St. Louis, from the special committee on the epidemics of Missouri, Illinois, Iowa, and Wisconsin, read an abstract of a report, which was ordered to be printed.

Dr. White, of New York, from the committee to which was referred the subject of re-admitting members who have not paid the annual assessment in proper time, reported the following resolution, which was adopted:—

Resolved, That upon no permanent member who is not present at a meeting of the association shall be assessed the annual contribution, but no one shall be entitled to receive a copy of the printed transactions, unless he pay into the treasury a sum not less than the annual assessment paid by the delegates and permanent members in attendance, and that all the names of permanent members that have been left off the published list, be inserted in the next volume of transactions.

Resolved, That no assessment whatever be made against members by invitation, but that they also be entitled to a copy of the printed transactions, by paying the sum assessed upon delegates in attendance.

Dr. Sanford B. Hunt, of Buffalo, New York, from the committee on the Hygrometrical state of the Atmosphere in various localities and its influence on Health, submitted a report, pending the reading of which the hour of adjournment arrived. The association resolved to meet at 9 o'clock to-morrow morning.

At 4 o'clock in the afternoon, the delegates, to the number of some 400, were taken in twenty-five coaches to the Blockley Insane Hospital. At the entrance the guests were met by Dr. Kirkbride and several other citizens distinguished for their philanthropic zeal, and after passing through and carefully examining the many departments of this noble institution, they passed to lawns and grounds surrounding the buildings, and various exclamations of gratification were heard on all sides. The visit afforded to many of our guests from sections of our country widely remote, an opportunity seldom enjoyed of comparing notes in regard to their locations and other facts. The representation from all parts of the Union is large.

In the evening they were entertained, at their residences, by Drs. S. L. Hodge, G. W. Norris and F. Bache.

### Second Day.

The Association assembled at 9 o'clock yesterday morning. The minutes of the meeting of the preceding day were read and approved.

Dr. Condie moved that the secretaries of the Association be requested to afford every facility possible to the reporters of the public press to enable them to furnish full and accurate reports of the transactions.

Dr. Attlee of Lancaster, asked and received permission to make a statement. At the last meeting of the Association a resolution was passed, authorizing the appointment of a Committee to procure a suitable stone for the Association to contribute to the Washington Monument, at Washington City, D. C. The Committee had made an assessment of One Dollar upon each member to purchase the stone and pay for the sculpture. Dr. Pierson, of Salem, Mass., had recommended as a design for the sculpture, "Hippocrates refusing the bribe offered by the King of Persia, when the great father of medicine said. 'Tell your master that I am rich enough; that honor will not allow me to succor the enemies of Greece.'" Not long afterwards Dr. Pierson was killed at Norwalk.

The designation upon the stone was executed by a young man named John Augustus Beck, only twenty-two years of age. Eminent artists had said that they did not think there was another sculptor in America who would have performed that work. Mr. Beck had been encouraged to go to Italy for study, and it is probable that he will rank among the first sculptors of the world.

Dr. Atlee then appealed to the members to come forward and make up a handsome compensation for the young artist.

A resolution was offered, to the effect that no member should speak unless his name and residence were announced. Adopted.

Dr. Hays then read a list of delegates whose names had been registered since the calling of the roll on the previous day. The additional names makes the total number present at this meeting of the Association 473, being the largest meeting of the Association ever held.

The additional names are as follows:

LIST OF ADDITIONAL DELEGATES.

Milwaukee medical society—J K Bartlett.

Wisconsin state medical society—Clark G Pease.

Rush medical college, Ill—J V Z Blaney.

Ohio state medical society—T H Baker.

Wayne county medical society—W S Battle.

Permanent member from Alabama—S W Claxton.

Medical society of South Carolina—P C Gaillard.

South Carolina medical association—Armory Coffin.

North Carolina medical society—O F Manson.

State medical society of Virginia—P C Cooch; J C Cabell.

Member by invitation—P F Brown, of Virginia.

Distict of Columbia.—Medical association of District of Columbia—W J C Duhamel.

Path. society of Baltimore—F Donaldson.

Kent county medical society—Thomas C Kennon.

Permanent members—From Pennsylvania, Washington L Atlee, John D Ross, James Bryan, Wm Ashmead, John Lewman.

Philadelphia county medical society—John F Lamb, Lewis Rodman.

Cambria county medical society—R M F Jackson.

Lancaster city and county medical society—W S Thompson.

Montgomery county medical society—Wm Corson.

Schuylkill county medical society—James S Carpenter, George Halberstadt.

Gloucester county medical society—Charles F Clark.

Camden county district medical society—Isaac S Mulford.

Mercer county (N J) district medical society—John H P Phillips.

Hunterdon county (N Y) medical society—John Blane.

State medical society of New Jersey—Zachariah Read, B H Stratton.

Essex county (N J) medical society—Linden A Smith.

Cumberland county (N J) medical society—Enoch Fithian.

Permanent members from New York—P B Brooks, A Willard.

Bellevue Hospital of New York—Stephen Smith.

New York county medical society—John Shark, J Warren, Henry S Davis.

Albany medical college—Alden Marsh.

Wayne county medical socieiy—C G Pomeroy.

Oswego county medical society—Charles G Bacon.

New York medical society—John E Todd, Edson Carr.

New York academy of medicine—H Weeks Browu, E B Warner, Joseph Wooster, Charles Herschel, S P White, James O Pond, Jam s L Phelps, J P Batchelder.

N Y Pathological society—S S Purple, James C Hutchinson, Thos F Cook.

RHODE ISLAND.—R I medical society—Chas W Parson.

Providence medical association—Wm O Brown.

CONNECTICUT—New Haven county medical society—Chas H Lindley, Joel Canfield, S N Beardley.

Middlesex county medical association—Geo W Barker, Ira Hutchinson, E B Nye.

Middlesex central medical association—David Harrison.

Fairfield county medical society—Robert Hubbard.

Permanent member—Gideon L Platt.

MASSACHUSETTS—Massachusetts state medical society—Stephen Hare, John Green, Wm Workman, Jos Sargent, C C Holmes, E D Miller;

Boston society for medical improvement—John Homan;

Castleton medical college of Vermont—J Perkins;

New Hampshire state medical society—Isaiah Crosby;

The chairman of the committee on nominations reported the following nominations;

President—Geo W Wood, M D, of Pennsylvania;

Vice Presidents—Wm M Boling, of Alabama; Daniel Tilden, of Ohio; P Humphrey Storer, of Massachusetts; Grafton Tyler, of the District of Columbia;

Secretaries—Francis West, of Pennsylvania; R C Foster, of Tennessee;

Treasurer—Casper Wistar, of Pennsylvania;

Committee on Publication—Francis G Smith, of Pennsylvania, Chairman; Francis West, of do; R E Foster, of Tennessee; Samuel L Hollingsworth, of Pennsylvania; H S Askien, of Delaware.

The Committee also recommended Nashville, Tennessee, as the next place of holding the annual meeting.

The officers recommended were, on motion, approved by the Association.

On motion, a Committee was appointed to conduct the officers to their seats, and the latter took their seats amid much applause.

Dr. Wood, on taking the chair, said he was deeply sensible of the honor conferred by the appointment, and none the less from an impression that it was probably an exhibition of respect for the place where the Association was holding its meeting. Personally he had a deep sympathy with the purposes for the advancement of which the Association was established. He had devoted his past life to the advancement of these objects, and he would devote to the same the little that might remain. He was unaccustomed to presiding over such large assemblies, but he would endeavor to justify the appointment.

On motion, the thanks of the Association were tendered to the retiring President, Dr. Chas. A. Pope, of Missouri, for the able and impartial manner in which he had presided over the meetings of the Association.

It was moved that the next meeting of the Association be held at Washington, D.C.

It was moved to amend by substituting Nashville, Tenn., and then again to amend by substituting Detroit, Michigan.

It was moved that the resolution be laid upon the table. Agreed to.

The report of the Nominating Committee in regard to the place of meeting was then taken up.

Dr. Palmer of Chicago, advocated the claims of Detroit, Mich., and moved that that place be substituted for Nashville, Tenn. The question being taken up, the motion was adopted.

Dr. Foster, one of the newly elected Secretaries, tendered his resignation, which was, on motion, accepted.

Dr. Brodie of Michigan was then chosen to fill the vacancy.

The farther consideration of the report of Dr Sanford B. Hunt of New York, upon the Hygrometrical state of the atmosphere in various localities, was resumed.

Dr. Hunt read his report, which was very interesting, and was listened to with a great deal of attention. It abounded in facts and statistics, illustrating the influences of the changes in the atmosphere, particularly with reference to epidemics, and deduced several laws as governing a certain class of epidemics.

On motion, the report was referred to the Committee on Publication:

Dr. Frank H. Hamilton, of Buffalo, N. Y., then submitted a report upon the Frequency of Deformities in Fractures. The report was accompanied with voluminous statistics. The main object of the report was the demonstration of the impossibility of uniting a fractured femur without shortening the limb.

Dr. Hamilton said he had a word to say, but not in connection with the report. It was neceessary to do something to arrest the frequency of the prosecutions for mal-practice. The frequency of these prosecutions no longer surprised the members of the profession. They had become familiar. What occasions this frequency? Is it because there are jealous and designing men among the profession? There are a few, no doubt. But, upon the whole, no profession stands by itself as well as the medical profession. Young lawyers might have something to do with encouraging the prosecutions; but this would not account satisfactorily for the evil complained of The speaker thought the reason could be found in the imperfections of the art, and the reluctance of the profession to admit those imperfections. Members would assure the world, for instance, that a fractured femur could be united without shortening the limb—which was simply impossible. He trusted that the profession would be wiser in future, and acknowledge that they could not perform impossibilities. Even that city of medical science, Philadelphia, had not produced a book which could instruct physicians how to unite a fractured femur without shortening the limb.

The report was referred to the Committee on Publications.

Dr. Charles Hooker, of New Haven Conn , submitted a report "Diet for the Sick." The report lays down laws for the government of diet under various diseases, and specifies particular articles which may be given with benefit. Referred to the Committee on Publications.

On motion, a resolution was adopted, returning the thanks of the Association to the retiring Vice President, Secretaries and Treasurer.

An invitation to visit the Central High School to-day, at noon, was accepted.

A resolution was adopted, returning thanks to the railroad companies that had given commutation tickets to members of the Medical Association.

On motion, the Association adjourned to Independence Hall.

*The Medical Association at Independence Hall.*—About five minutes past twelve o'clock, the members of the Association entered Independence Hall. A number of ladies were already present, and the Hall was soon crowded to its utmost extent. Detachments of police, under the command of Lieut. Bailey, prevented the pressure of the crowd outside.

Dr. Isaac Hays, on behalf of the Medical Association, introduced the members in the following address :

*Speech of Dr. Hays.*—I have the gratification of introducing to your Honor the Members of the American Medical Association —our National Medical Congress. This association was organized in our city, eight years ago, and has met annually since in the principal cities of the Union alternately. It is composed of delegates from the Medical Societies, Medical Colleges, Hospitals, and other Medical Institutions throughout our country. It was instituted with no selfish views—but to accomplish objects of the greatest importance to the public at large ; to improve the healing art, and increase its powers for alleviating human suffering. It strives to attain those ends by securing more complete and thorough courses of instruction to students, and by raising the standard of requirements of those admitted into the ranks of the profession; by investigating the causes of the diseases which prevail in certain localities, and by seeking the means by which these causes may be removed, or their effects counteracted; by collecting reliable histories of the different epidemics which, from time to time, spread over our country, and by endeavoring to discover some method of arresting their progress, by offering prizes for the most useful discoveries and improvements in medicine—and by promoting every measure tending to enlarge the boundaries of our science—increase the efficiency and augment the usefulness of our art.

In pursuance of these objects, the Association has assembled this year in our city, and we feel deeply grateful to you for your courtesy in inviting us to visit this place, so venerated by every American heart.

The philanthropy of our profession is not restricted to the cure of disease—it has a larger range, it embraces within its scope whatever tends to the improvement of our mental or moral condition, or even to confer the greatest good upon the largest number. Hence the profession have never been indifferent to national objects—on the contrary, they flatter themselves that they have not been behind any other class of the community in patriotism, but that they have been always prompt to serve their country, not only in their professional capacity, but also by their counsel in the Cabinet, and even by taking up arms for her defence in the field.

I need not remind your honor that the most illustrious physician of his day, in this country, the late Dr. Rush, was a member of the Congress of '76, and affixed his name to the Declaration of Independence; or that Gen. Joseph Warren, one of the most eminent physicians of Boston, contributed by his eloquence to rouse his townsmen to resist the oppressive acts of the mother country, and shed his life's blood on Bunker Hill in defence of liberty—or that his younger brother, Dr. John Warren, animated by the same spirit, volunteered as a private soldier, or of other examples of a similar character. But I may assure you that the spirit which animated our ancestors, if it appear dormant, is not extinct in our bosoms, and that while standing on this spot—the shrine sacred to Human Liberty—we experience feelings akin to those which the inspired Law-Giver must have felt when he heard the voice calling to him out of the midst of the burning bush: "Put off thy shoes from off thy feet, for the place whereon thou standest is holy ground."

To this his honor, Mayor Conrad, responded in the following truly eloquent speech:

*Reply of Mayor Conrad.*—Mr. Chairman of the Committee of Arrangement, I thank you in the name of the community which

I have the honor to represent, for your eloquent introduction of our friends to the authorities of the city, and to this the Hall of Independence.

Gentlemen of the American Medical Association, I am proud of the privilege of extending to you, in the name of the government and of the people, of Philadelphia, a most cordial welcome.

I bid you welcome to our city—a city which, deriving a cherished distinction from the profession which you adorn, is eager, now and ever, to requite it in her tribute of respect for its professors. I welcome you to our people, whose intercourse for many a year with you or your brethren, has inspired a feeling which, reserved as we are sometimes said to be, will, I doubt not, burst into earnest and unambiguous expression before you leave us.

I welcome you, gentlemen, to this Hall, but not as strangers, or the sons of strangers—for it is your own. As the temple and territory of Delphos, in the wildest domestic perturbations of Greece, afforded one sacred area over which the cloud of discord never gathered, one altar whose worship was never invaded, this spot, consecrated to our common American glory, knows no lines of latitude, and belongs, in truth, no more to us, whose peculiar privilege it is to inherit its guardianship, than to our brothers—*to you.* In coming hither, therefore, you *come home.* These precincts have been hallowed for all time by the heroic virtues of your and our fathers. This is the fountain from which the living waters of American liberty were first drawn, and it is, therefore, most sacred—(wo to the generation in which it ceases to be sacred!)—but, like the well of the Patriarch, all the tribes of Liberty's Israel own here an equal right, and own here an equal homage.

In no sense, then, can I greet you as strangers; for yours are names familiar to every American proud of the science of his country, and those who are united, by this Association, in a cause so lofty as that eloquently characterized by your chairman, may not only claim the universal and acknowledged privileges of the republic of minds, but the rights of a nearer and dearer charter, the brotherhood of beneficence—the kindred claims of noble hearts, knit in the highest and holiest of human aspirations. In this spirit, with the most fervent and fraternal sentiments of respect and regard, I greet and welcome you.

You are right, Mr. Chairman, in claiming, amid the associations which hallow these precincts, a peculiar privilege for your profession—a profession which not only sprinkled, with the first blood of the Revolution, the highest altar upon which valor vowed and dedicated our country to freedom—I refer, as you have referred, to Dr. Warren and Bunker Hill—but which, in every struggle for the enlargement and enlightenment of human destinies, has been eminently distinguished for courage, zeal and fidelity to the rights of man. You have, therefore, a peculiar right to kindred here, and have that claim allowed; and within these walls, which witnessed the zeal of Rush, it would be a treason to virtue to forget that one of the lights of your profession shed glory upon the solemn debates of this hall, and was foremost among those that bade yonder bell (preserved and devoted to the veneration of posterity) with its iron tongue to "proclaim liberty throughout all the land to all the inhabitants thereof."

It is the glorious peculiarity of your profession that while ambition, in its ordinary and most applauded paths, plays the part of the *Destroyer*, and wins glory at the expense of human life and happiness, you and yours, with a more exalted civilization and a nobler heroism, have ever sought to save. Next to the highest of all human courage—if, indeed, it be merely human—that of the martyrs of religious truth, the courage of the physician, whether on the battle-field or in the lazar-house, the courage of science and humanity is the most sublime, and best entitled to the *clarum et venerabile nomen*. The vulgar courage of the warrior under the base stimulus of passion or the low greed of applause, can hardly be compared to the noble intrepidity of the surgeon, who gleans, in the ruthless and red-handed reaper's path, the leavings of the battle; and still less with the hero of the hospital, who encounters the grim antagonist in the horrid silence and gloom of the pestilence. Imagination can hardly embody an instance of human courage and virtue more sublime and unearthly than that of the physician, who, in the midnight of a plague-stricken city, midst the fœtid solitude of its alleys, and entering the devoted hovel of the wretched, ministers, while only pestilence, and misery, and death, and God look on, to the perishing. I need not step from this spot to grasp the hand of many a hero who claims no laurel—many a noble philanthropist whose sacred labors, in scenes like these, have been unmarked, save by the eye that never slumbers, and remembered only by him who alone can reward.

To such a profession, one venerable from its antiquity, noble from the grandeur of its objects, illustrious from its achievements, and which demands every aid and energy of genius and science of head and heart that dignifies the race, it is not strange that, go where it may, a ready homage greets and a ready blessing attends it. In our own city, all that is noble in patriotism, all that is exalted in science, all that is bright and beautiful in the arts that refine society, all that is lovely, and cherished and holy in private life, combine to render the profession sacred and dear to us.

There are few living to whom some one death in the past is not the sole event and solitary memory of the survivor's life—to him a lonely pyramid in the melancholy desert; and to such a mind and memory the debt of the death-bed, where science, rendered holy by its office, ministered, though never paid, is never repudiated. I never knew a good man, still less a good woman, who had not such a debt—a debt which bankrupt gratitude cherished with its holiest affections and devoutest memories.

In these times, when the omnipotence ef associated effort is invoked for so much that is of dubious merit, it is a gratifying spectacle to behold the enlightened professors of the most exalted of all arts—men sage and grave, unselfish and unaspiring, forsaking the home to which they are bound by the affections and afflictions of thousands, by wealth, and fame, and influence, to wander weary away upon a pilgrimage of hundreds of leagues, in the cause and interests of the human family, its security, its health, and its happiness. For more than ten years, the representatives of your profession have gathered in Convention. What other body of our citizeus have made a like effort—a like sacrifice? Selected from the most eminent of the profession, the delegates have been men whose years, like their virtues, were many. How difficult must have been to them the effort to burst through the bonds of a relying and clinging practice! How great the labor, and how heavy the sacrifice! They have already visited, in this duty, the cities of every section of our wide country. How many have fallen by the wayside? How many martyrs could you not thus number in this cause? How many of the good and great of the profession have, in these benevolent pilgrimages, joined the ranks of the thousands who have sacrificed themselves at the requisitions to duty, as recognized and enforced by your self-imposed laws—joining the dead in order to aid the living? The epitaph of the Spartans at Thermopylæ might well commemorate the virtues and the fate of these martyrs. But if the cost has been great, the results have been commensurate.

Of the professional advantages attained, though I know them to be invaluable, I will not presume to speak; but I may be permitted to state, as health is the most important subject of municipal provision and care, that the Transactions of the Association, which I have examined with great interest, comprise much that merits the attention and will reward the respectful consideration of the municipal governments of the Union.

It is natural that Philadelphia should feel, as she does feel, a profound interest in the cause of medical education in this country. She cannot of course, forget that it was here that the first medical college was established in this country; that its merits and success extorted a reluctant trans-atlantic tribute of admiration, and that progressing rapidly but wisely, it achieved and maintained an equality with the most celebrated institutions of the old world. As the cause of medical education has expanded, and institutions worthy of the cause and country have sprung up, each triumph thus attained has been regarded here as the successful outbursting of an off-shoot from the primary effort; and Philadelphia, while rejoicing at the expansion and elevation of medical education throughout the land, has almost fancied—so earnest is her interest in medical education—that she had a right to indulge a parental pride in all that advances that interest.

These genial feelings have been maintained, in all their early and fervid freshness, by constant intercourse with all sections of our country. The ingenious and gallant youths that have come here for medical instruction have, in their unstudied intercourse exhibited the character of their respective States in a light so generous and exalted as to win our affections not only for themselves but for the communities and States which could exult in them as their own. Winter after winter, we have had hundreds of these noble young spirits among us here. And let me remark that, rigorous as I am said to be in the administration of the law, I have yet to know the first occasion to rebuke, much less to punish a medical student. We have found them as gentle and decorous in their deportment as they are exalted in their aspirations, and had Philadelphia—eminently catholic in her affection for sister communities—needed a lesson of love and loyalty, these noble young missionaries would have taught it. This interchange of sympathies has endured for the third of a century, (may it last forever.) Her youths who formerly bore those sentiments to the remote sections of our republic, stand before me now as the revered sages and ornaments of their profession, meeting here the evidences of a reputation which had preceded them and has long been cherished by us. And who can tell what have been the results of this kindly interchange of kindly feelings? It has doubtless been felt in every commercial, social and political relation of life, correcting the prejudices, harmonizing the discords and subduing the dangers of our common country.

We realize these facts. We recognize in the members of an enlightened profession like yours, so many patriots and philanthropists engaged in the great and general interests of the human race; and apart from the mere scientific acquisitions of your annual meetings, we perceive, in them, results auspicious to all that we cherish, all that is kindly, forbearing and conservative between man and man, party and party, state and state, section and section; and so regarding them, we hail and greet you with a welcome as sincere and cordial as the heart can forge or the tongue can utter.

The members spent some time in viewing the pictures and relics in the hall, and then returned to the Musical Fund Hall.

*The Afternoon Session.*—Two invitations, one to visit Langenheim's Photographic Academy, and one from the Board of Health, to visit the City Hospital and the Lazaretto, were accepted.

On motion of Dr. N S. Davis, the thanks of the Association were tendered to the Mayor of Philadelphia for the very cordial manner in which he had received the American Medical Association at Independence Hall, and that a copy of his speech be published in connection with the proceedings of the Association.

On motion, the rules were suspended, and Dr. Thompson, of Delaware, offered the following preamble and resolutions:

Whereas, That as few subjects of greater interest and importance could be presented to the consideration of the American Medical Association, now representing most of the States and Territories of the Union, than the attainment of a correct Medical Topography of each, with a history of its prevailing fevers, and the most successful treatment of the same, therefore be it

Resolved—That with this view and conviction, this Association now appoint a special commission from each State and Territory, represented of ——— members, whose duty it shall be to report upon its medical topography, epidemic fevers, and the most successful treatment thereof, and that the same shall continue to hold their office for three years.

Resolved—That in the appointment of gentlemen of education and experience in their own State, we have the best guarantee that the important objects we seek will be most satisfactorily accomplished, and the profession as well as the public interest will thereby be better served.

Resolved—That the Committee appointed by the Association, at its annual meeting in Charleston, S.C., for the same purpose, be, and the same are hereby discharged.

Dr. Thompson moved that the resolutions be made the special order for to-day, at 10 o'clock A.M. Agreed to.

The Committee on Publication submitted a report, from which we learn that the seventh volume of the proceedings of the Association was issued last November, 1,000 copies being published, at an expense of $1.806 42; 781 copies have been sold or furnished to members of the Association; 31 were given to editors of medical journals; and 184 remain on hand. Two resolutions appended to the report, in regard to restricting the furnishing of the copies of the proceedings, were adopted.

The Treasurer submitted his annual report, from which we gather the following facts:

| | | |
|---|---|---|
| Balance in the Treasury at last account, | - | $273 99 |
| Assessment and sales of Transactions, | - | 2722 31½ |
| Prize Fund, - - - - - - | - | 200 00 |
| Total, - - - - - | - | $3216 30½ |
| Balance in Treasurer's hands at present, | - | 1115 26 |

Dr. Condie, from the Committee on Publication, read a report upon certain charges made against the Committee in regard to the publication of the proceedings, and a charge made against Dr. Meigs, of this city, of making profit by publishing the material of the Association as his own work; and also upon the question whether the Association should publish its own proceedings, or entrust it to a regular bookseller. The charges against the Committee were that they had delayed the publication of the last volume of the proceedings, and had excluded several papers that were preeented at the meeting of the Association. Both these charges were disproved, and Dr. Meigs was exonerated from the charge made against him. The Committee decided that it was not expedient for the Association to publish its own proceedings. Dr. Condie asked that the report be entered upon the journal

A resolution was offered, thanking the Committee on Publication for the faithful performance of their arduous duties, and expressing the satisfaction of the Association therewith.

Dr. Condie stated that the Committee on Publication cared but little for the charges made against them; but they desired to do justice to a man who stood very high in reputation in Philadelphia, and who felt himself aggrieved by slanderous accusations. He withdrew the request that the report of the Committee be entered upon the minutes.

A resolution was adopted, appropriating the sum of $1,000, to pay for the stone for the Washington Monument.

Dr. Duhamel offered a preamble and resolutions returning the thanks of the Association to those Senators and Representatives who took an interest in procuring the passage of the Quarantine bill, the object of which is the better prevention of the introduction of diseases into the country.

The resolutions were adopted.

The Secretary read a paper from Dr. Wm. H. Byford, of Evansville, Indiana, upon Scrofula. This paper gives a precise account of the disease, its varieties, causes, effects, and treatment. Referred to the Committee on Publications.

Dr. N. .S Davis of Chicago, Ill., read a report upon "The Nutritive Qualities of Milk, and the Influence produced thereon by

Pregnancy and Menstruation in the Human Female, and by Pregnancy in the Cow ; and also on the question whether there is not some mode by which the nutritive constituents of milk can be preserved in their purity and sweetness, and furnished to the inhabitants of cities in such quantities as to supersede the present defective and often unwholesome supply." This a very able paper, possessing more general interest than most other reports. The various methods of preserving milk are all investigated and explained, and the preference given to that discovered by a gentleman of Dutchess Co., New York. By this method milk had been preserved for twelve months, with all its nutritive qualities. The solidified milk produced by that gentleman, was decided to be the article that had long been a desideratum. Referred to the Committee on Publication. The committee was continued.

The hour for adjournment having arrived, on motion, the Association adjourned.

According to previous arrangement, the members of the American Medical Association and others, to the number of about 500 persons, visited Girard College and Fairmount. The party entered coaches at the Musical Fund Hall, about 4 o'clock, and proceeded first to Girard College. In the hall of the hall of the Directors, in the main building, Dr. Emerson, of this city, introduced the members of the Association to President Allen. He said they were congregated from all parts of the Union they were much gratified at the privilege of visiting the institution.

President Allen replied briefly, extending a cordial welcome to the members of the Association. He rejoiced to have the pleasure of meeting so many members of the noble profession of medicine. He had not anticipated being called upon to make a formal speech. He could only say that it gave him much pleasure to see them at the college—an institution founded by one of the noblest charities the world has ever known. The various departments of the College were now open to the inspection of the Association. The schools had been kept in session longer than usual, in order to allow the visitors to see the pupils.

The members of the Association then passed up stairs to the schoolrooms, where they beheld the boys, 310 in number, seated and under the charge of their female teachers. They presented a neat and interesting appearance. From the schoolrooms, the party proceeded to the roof of the College, from which they obtained a glorious view of the vast city and the beautiful country around. The various apartments in the main building, and the others inspected, and the tasteful grounds were traversed; and then the party once more entered the coaches to go to Fairmount.

Fairmount was reached just before sunset. The afternoon was genial and pleasant, and everything at Fairmount wore a fresh and verdant aspect. The various portions of the ground, the reservoirs, etc., were visited, and then the party returned to the city.

---

THIRD DAY.

The Association met at nine o'clock in the morning, pursuant to adjournment. Dr. Wood in the chair. The minutes of the proceedings of the last meeting having been read by Dr. Francis West, Secretary, the President stated that there was no rule or mode for the appointing of the Committee on Prize Essays, and suggested that the subject be referred to the Nominating Committee. A motion to this effect was agreed to.

A letter was read from Dr. Rayburn, of Missouri, suggesting that the large district in Missouri be divided into two parts. The district is seven hundred miles north and south, and four hundred miles east and west. The duties in so large a district as this cannot be easily fulfilled by one chairman. By dividing the district, the reports will be more satisfactory than at the present time. The Doctor tendered his resignation.

Dr. Watson moved that the letter be referred to the Nominating Committee, and the resignation of Dr. Rayburn be accepted.

The President ruled that part of the motion relating to the reference out of order, as it conflicts with the resolutions made the order of the day at ten o'clock.

The resignation of Dr. Rayburn was accepted.

Dr. Isaac Hays, from the committee of arrangements, stated that he was not ready to make a report at the present time of the full list, but would do so at a later hour in the day. He announced that 510 delegates were now enrolled, and the duties of the committee have become more arduous. The Doctor read invitations to visit St. Stephen's Church, to look at the Byrd monument. Accepted.

Thanks were tendered to the Rev. Dr. Ducachet for his kindness in extending an invitation.

The Association now proceeded to the consideration of the reports of the various committees.

Dr. E. B. Haskins, of Clarksville, Tenn., to whom was referred the subject of "Microscopical Investigation of Malignant Tumors," reported that he was unable to submit a report, in consequence of not having had the necessary opportunities. He was excused.

The committee on nominations reported the following committees for the present year:

On Prize Essays Dr. A. B. Palmer, of Michigan; Samuel Denton, do.; A. R. Terry, do.; Abraham Sagar, do.; J. H. Douglass, do.; Corkdon La Ford, do.; E. Andrews, do.

On Arrangements—Dr. Zino Pitcher, Detroit, Moses Gunn, do.; G. B. Russell, do.; N. S. Leland, do.; M. Stewart, do.; P. Klein, do.; J. A. Brown, do.

On Medical Literature—Drs P. C. Gillard, S. Carolina; N.P. Monroe, Me.; James Cooper, Del.; R. Hills, O.; A. Coffin, S. Carolina.

On plans of organization for State and County Societies—Drs. A. B. Palmer, of Michigan; N. B. Ives, of Connecticut; E. B. Haskins, of Tennessee; Chas. Woodward, Ohio; Josiah Crosby, New Hampshire.

On Medical Education.—Drs. J. Berrien Lindsley, of Tennessee; J. B. Flint, of Kentucky; P. H. Cabell, of Ala.; Geo, Hayward, Mass.; E. B, Smith, Missouri

The report of the Committee was unanimously accepted.

Dr. Frank Hamilton, of Buffalo, New York, made an additional report on the subject of fractured clavicles. It was listened to with marked attention. At the concluson of reading the report, the Dr. expressed an earnest hope that the Managers of the Pennsylvania Hospital would be more exact in making up their reports of the statistics of fractured clavicles, so that we may all be enabled to judge of the merit of the instrument, which has been used in such cases in that Institution, for thirty years. He had known a surgeon to be mulcted into heavy damages for using that instrument in a case of fractured clavicle, because he could not accomplish all that he supposed he could by using it. He did not wish to speak harshly, but at the same time he called again upon the managers of that Institution to be more exact in their statistics on the subject of fractured clavicles, so that the medical faculty may be prevented from being a grand Insurance Company for the whole world.

The following resolutions being the order of the day, were read and considered:

Whereas, That as few subjects of greater interest and importance could be presented to the consideration of the American Medical Association, now representing most of the States and Territories of the Union, than the attainment of a correct Medical Topography of each, with a history of its prevailing fevers, and the most successful treatment of the same, therefore be it

Resolved—That with this view and conviction, this Association now appoint a special commission from each State and Territory, represented of ——— members. whose duty it shall be to report upon its medical topography, epidemic fevers, and the most successful treatment thereof, and that the same shall continue to hold their office for three years.

Resolved—That in the appointment of gentlemen of education and experience in their own State, we have the best guarantee that the important objects we seek will be most satisfactorily accomplished, and the profession as well as the public interest will thereby be better served.

Resolved, That the Committees heretofore appointed by this Association, at its session in Charleston, for a similar object, be, and at the same time are hereby discharged.

On motion of Dr. J L, Atlee, the Hon. Ellis Lewis Chief Justice of Pennsylvania, (being in the room,) was unanimously invited to a seat on the platform.

Dr. Askew offered the following as an additional resolution :

Resolved, That all reports on the Medical Topography and prevailing diseases of States, shall, to entitle them to be received by this Association and published in the proceedings, be first approved by the Medical Societies of the State or Territory where such Societies exist, and to which State or Territory such report refers.

Dr. J. G. Orton, of New York, offered the following additional resolutions :

Resolved, That each County Medical Society, or in parts of the country where such have not been established in any duly organized Medical Association, be invited to amend their Constitution by attaching thereto the following article :

"It shall be the duty of each member of this Society to keep a faithful record of the diseases which may fall under his observation during each month, according to the classification adopted by this Convention, in May 1847, stating the age and sex, occupation and nativity of the patient, the average duration of the disease, and finally their recovery or death, and to report the same in writing to the Secretary, on or before the 1st of February of each year, who shall transmit a digest therof to the State Medical Society, and also the appropriate committee appointed by the American Medical Association for its reception..

Resolved, That each incorporated Hospital, Infirmary and Asylum be invited to furnish a copy of their annual reports, for the use of the committees of their respective State.

Resolved, That the State Committee appointed by this Assotion, to report on the prevailing diseases of their respective locality, shall receive and arrange a digest of the reports transmitted to them by the Secretaries of the various county Societies, and to report the same at the annual meeting of this Association.

Resolved, That the first day of January be the time fixed at which the object of these resolutions shall be carried into effect, and that the several County Societies and Associations be requested to amend their Constitution as heretofore recommended, at as early a date as practicable, and to report to the State Committee their willingness or unwillingness to acquiesce in the request of this Association.

A motion was made that the whole subject be referred to the Nominating Committee.

The previous question was called, and being sustained, the motion to refer prevailed unanimously.

Dr. Thompson of Delaware expressed a hope that the Committee would go into immediate session, so that a report might be made as soon as possible.

Letters from Drs Sutton and Denner, on the subject of epidemics, were referred to the Committee on Nominations:

Dr. D. Francis Condie, to whom was referred the subject of tubercular disease, stated that he had been engaged for a period of three years in examining and arranging a report on this subject. The report will occupy at least five hundred pages, and one reason which has caused it to swell to so great an extent, is, that he found it necessary to contradict a large number of statements and reports on the subject. He hoped that the Association would excuse him in not being quite ready with the report. He had prepared a digest of its contents, which in fact is merely an index to the whole, and would not of itself repay for the time used in reading it. He expressed a desire that the Association would excuse him.

A motion was made that Dr. Condie do just as he please, without any obligation he may consider himself under to the Association. The motion was agreed to.

The Committee on diseases of the throat, were continued for another year.

The committee on the subject of Dysentery, submitted a report, which was referred to the committee for publication.

A brief report from the committee on Hydrophobia, was read and referred to the committee for publication.

The following named, additional delegates were enrolled.

RDODE ISLAND.

Rhode Island state medical society.—David King.

MASSACHUSETTS.

Massachusetts medical society.—Jno. Ellis Blake Benj. Cutter Francis Leland.

Boston society for Medical observation—Luther Parks, Jr.

Permanent members—George Choate. Levi Folsom.

VERMONT.

Charles Clark.

NEW HAMPSHIRE.

Grafton district medical society—H B Brown.

New Hampshire state medical society—Fred. Boyden.

MAINE.

Medical school of Maine—E M Peaslee.

MICHIGAN.

Permanent members—Aaron L Leland, Isaac Paddock.

Michigan state medical society—S M Axtille,

OHIO.

Commeraial hospital, Cincinnati—A Evans.

Miami medical college—C G Comegys.

Medical college of Ohio—Thos. Wood.

Sterling medical college—S M Smith, John Dawson.

Cincinnati medical society—J J F Holston.

Member by invitation—Caleb Jones.

ALABAMA.

A. Denny.

SOUTH CAROLINA.

South Carolina medical society—Octavius White.

Permanent members—R A Kinlock, H W De Saussure.

NORTH CAROLINA.

North Carolina state medical society—N J Pitman, Chas. F. Denny, J R Thompson.

Edgecombe medical society—B W Marbrey.

VIRGINIA.

Permanent member—W B Cochran.

State medical society—J M Hurst.

Prince Edward county medical society—A S Dillon.

DISTRICT OF COLUMBIA.

Georgetown medical college and medical association of district of Columbia—J E Morgan.

DELAWARE.

New Castle county medical society—John P Wales,

Delaware state medical society—R R Porter.

MARYLAND.

Maryland Medical Chirurgical Faculty—Christian Johnson, Charles Frick, John K Sappinton, J Gilman, S R Handy, W B Crane, T E B Hintyre, George W Lawrence, Walter F Belt, John F Monmonier.

Baltimore Medical and Surgical Society—D A O'Donnell.

Baltimore Pathological Society—William H Davis, William Riley.

PENNSYLVANIA.

Philadelphia Association for Medical Instruction—Addinell Henson.

Bucks County Medical Society—O P James.

Pennsylvania Hospital—Wm. Pepper.

York County Medical Society—Jams W Kerr.

Philadelphia Medico-Chirurgical Society—Henry Y Smith.

U. S. Navy—G R B Horner.

Allegheny County Medical Society—A M Pollock.

Chester County Medical Society—Wilmer Worthington, Alex. K Gaston, S A Ogier.

Vorthampton county medical society—Pobert E James, Edward Swift.

State medical society—John J Koehler, J M Couper, W W Townsend.

Lancaster county medical society—John Ream.

Lancaster county hospital—J Aug. Ehler.

Permanent members—Anthony Heger, Moreton Stille, George Fox, Lawrence Turnbull, Thomas S Spencer, Trail Green, Wm. J Wilson, W G Wimley.

Huntington county medical society—J H Dorsey.

NEW JERSEY.

Essex district medical society—George R Chatwood.

Permanent members—Quinton Gibson, J B Munn.

Mercer connty district medical society—Thos. J Corson.

Warren county district medical society—P F Brakely.

NEW YORK.

New York academy of medicine—J P Garrish, Pliny Earle, W N Blakeman, Lewis A Sayre.

King's county medical society—T Anderson Wade.

German medico chirurgical society—Louis Bauer.

Statistical society, N. Y.—D S Conant.

Monroe county medical association—Henry W Dean.

Geneva medical college.—F. Hyde.

Permanent members—Harvey Jewett, James M Austin.

Wayne county medical society—T P H Deming.

West Chester county medical society—James D Trask.

New York medical association—Jos. A Monell.

New York Ophthalmic hospital—Mark Stephenson.

Nhw York medical society—H D Balkley.

College of physicians and surgeons, N. Y.—Alonzo Clark.

MISSOURI.

St. Louis medical society—E F Smith.

KENTUCKY.

Permanent member—Walter A Norwood.

CONNECTICUT.

Fairfield county medical society—David S Barr.

New Haven medical association—Thomas H Totten, Benj. H Catlin, John R Downes.

Litchfield county medical society—Wm. W Welsh.

Hartford hospital—Myron W Wilson.

Norwich county medical association—Ashbel Woodward.

Dr. H ys stated that the whole numbers amounted to five hundred and twenty. The hour of recess having arrived, the association separated.

AFTERNOON SESSION.

The Association re-assembled at 1 o'clock.

Dr. Muzzy of Ohio, read an interesting report on the use of alcohol, which, on motion of Dr Condie, was referred to the Committee on Publication

An invitation was received from the Principal of the Pennsylvania Institute for the training of feeble minded children.

The Nominating Committee, to whom was referred the resolutions providing for the appointing of a committee on Medical Topography, referred the same back without material ameudment, and recommended the following named as the committee:

Maine—J. C. Weston, of Bangor.
New Hampshire—Edmund R. Peaslee, Dartmouth College.
Vermont—Joseph Perkins, of Castleton.
Rhode Island—Joseph Mauran, Providence.
Connecticut—Charles Hooker, New Haven.
Massachusetts—George C. Shattuck, Boston.
New York—Joseph M. Smith.
New Jersey—Lyndon A. Smith, Newark.
Pennsylvania---Jacob M. Gummill, Huntington County.
Delaware---James W. Thompson, Wilmington.
Maryland---Peregrine Wroth, Chestertown.
Georgia- -John F. Posey, Savannah.
Virginia---P. F. Peebles, Petersburg.
District of Columbia---Thomas Miller, Washington.
South Carolina---D. J. Cain, Charleston.
North Carolina---O. F. Manson, ———
Kentucky—Wm. L. Sutton, Georgetown.
Tennessee—E. B. Haskins, Clarkeville.
Louisiana—E. J. Fenner, New Orleans.
Minnesota—J. H. Murphy, St. Anthony Falls.
Ohio—G. Menderhall, Cincinnati.

Mississippi—T. J. Grafton, Rodney.
Missouri—S. B. Alleyne.
Michigan—J. H. Beech, Cold Water.
Alabama.—S. W. Clanton, Warsaw.
Illinois—John Evans, Chicago.
Indiana---Vierling Kersey, Milton, Wayne co.
Wisconsin---Alfred L. Castleman, Delafield.
Iowa---E. A. Arnold, Davenport.
U. S. Navy---Thomas Dillard, Philadelphia.
U. S. Army---Clement A. Finley.

Dr. Thompson, who offered the original resolutions, begged as a personal favor, to be excused, and that the subject be referred to the delegate from his state. The Association would not excuse him.

The same committee, to which was referred so much of the President's address as relates to the next place of meeting, reported that it was inexpedient to change the place from that agreed upon by the Asssociation.

The committee further reported the following named special committees on the subjects named.

On Puerperal Fever and its communicability—S. W. Noble, Leroy, McLean co., Ill.

On the progress of general and descriptive anatomy—J. W. Freer, Chicago, Ill.

On the diversity of the venereal poison.—Dr. T. G. Richardson, of Louisville, Ky.

On the effect of mercury on the living animal tissues—J. B. Coleman, New Jersey.

On the best mode of rendering the medical patronage of the national government tributary to the honor and improvement of the profession—J. B. Flint, Kentucky.

On the treatment best adapted to each variety of cataract, with the method of operation, place of election, time, age, &c.,—Mark Stephen.

On the causes of *infant mortality* in large cities, the sources of its increase, and the means for its dimunition.—D. Meridith Reese, N. York.

On the causes of the impulse of the heart, and the agencies which influence it in health and disease—John W. Corson, N. Y.

On sanitary police of cities--James M. Newman, Buffalo, N. Y.

On the native substitutes for cinchona indigenous to the southern states—P. H. Cabell, Ala.

On endemic tetanus—J. Knight, New Haven.

On strychnia---its chemical and toxicological properties---Dr. Lewis H. Steiner, Washington, D. C.

On tracheotomy in epilepsy---Ashbury Evans, Covington, Ky.

On treatment of the cholera---J. Taylor Bradford, of Augusta, Kentucky.

On malignant periodic fevers---Charles Quales Chandler, of Rockport, Me.

On the execretions as an index to the organic changes going on in the system---H. A. Johnson, Chicago.

On microscopical investigations of malignant tumors.---Henry J. Bigelow, Boston, Mass.

On the statistics of the calculaus disease and the operations therefor---J. S. Carpenter, Pennsylvania.

On the treatment and curability of reducible hernia---J. S. Carpenter, Pennsylvania.

On the best treatment of cholera infantum—A. J. Fuller, Maine.

On injuries of the joints— Wm. B. Page.

On the treatment and curability of reducible hernia.---D. Carpenter, Pennsylvania.

On the statistics of Mortality in the United States---Wilson Jewell, Philadelphia.

On motion, Dr. Anderson was continued as chairman of the committee on Education.

A resolution relating to the registration of births, deaths and marriages, was offered, and referred to the committee on nominations. The association adjourned.

VISIT TO THE ALMSHOUSE.

In the afternoon the association visited the Philadelphia Hospital and Almshouse, at Blockley, Twenty-fourth ward, at the entrance of which they were met by several of the Guardians of the poor, and were ushered into the board room, where Dr Jewell, of this city, introduced the visitors to Frederick M. Adams, Esq., President of the Board, who in return, said:

Gentlemen of the American Medical Association and of the committee of arrangement: As the presiding officer of the Board of Guardians of the poor of the city of Philadelphia, the pleasing duty of welcoming you on behalf of the board devolves upon me, which, gentlemen permit me to do most heartily. It is not often that the Guardians have an opportunity afforded them of extending an invitation to such a distinguished scientific body---it is seldom they are enabled to proffer an invitation, the acceptance of which is so likely to be attended with beneficial results to humanity.

Permit me to say, it is not the less gratifying to them. In conclusion, gentlemen, trusting that the short time you may be enabled to devote to an examination of such objects as may engage your attention in the institution, may be productive of interest to you, I, in behalf of the Board of Guardians, greet you, and extend a cordial welcome. Allow me to introduce to you, Dr. A.

B. Campbell, the chief resident physician, and his several Assistants.

The association preceded by Dr. Campbell and the assistant physicians of the institution, visited the many wards, and expressed much appropriation of the many departments, they were ushered into a hall, where a slight collation was set out. The guests after refreshing themselves returned to the city, apparently well pleased with the visit. In the evening they were entertained the residences of Dr. Samuel Jackson, J. Pancoast, Henry Hartshorne, and Mr. Isaac Lee.

---

FOURTH DAY.

The association was assembled yesterday morning, pursuant to adjournment. Dr. Wood in the chair.

Dr. Isaac Hays, from the committee of arrangements, reported the following additional delegates, making a total number of five hundred and twenty-three.

MASSACHUSETTS.

Massachusetts General Hospital—J. Mason Warren.

NEW YORK.

New York pathological society—F. C. Frennell.

PENNSYLVANIA.

Lebanon county medical society—William Moore Guilford.

Dr. Atlee offered a resolution that Dr. Breckenbridge's report on medical literature be referred to a special committee, to be read, and if approved by said committee, then to be referred to the committe on publication. The motion was lost.

A motion was made and adopted, that the committee on medical literature be continued to report at the next meeting of the association.

Dr. Haywood, of Boston, offered the following, which was adopted unanimously:

*Resolved*, That the thanks of this association be presented to his honor the mayor, and the other officers of the city government, and the citizens and physicians of the city of Philadelphia, for their kind and continued attention, and munificent hospitality to the members of this association during the present session.

Duty L. Atlee, chairman of the special committee, to whom was referred, at the last annual meeting, the paper of Dr. Phelps "On Religion an element in medicine," &c., &c., reported that the paper was ably written and creditable to the author. But on account of its length and the fullness of our adopted code of ethics, was not expedient to publish it in the transactions of the association. The report was accepted and its recommendations adopted.

Dr. Isaac Hays, from the committee of arrangements, reported the name of Dr. Wm. H. Wattson, of Bradford county, Pa., as a member by invitation. The report was adopted.

A vote of thanks was tendered to the managers of the academy of fine arts, for their liberal offer to take charge of the stone for the Washington Monument, until its delivery at Washington.

The following amendments to the constitution was offered, and under the rules laid over until the next annual meeting of the association.

" Any permanent member who shall not pay for the published transactions for three successive years, shall be considered as withdrawn."

Dr. N. S. Davis, of Chicago, Ill, submitted the following preamble and resolution:

*Whereas*, The present mode of conducting the annual meetings of the association affords but little opportunity for the discussion of strictly scientific questions and papers, and

*Whereas*, This has been regarded as a serious defect in the operation of our organization, impairing its scientific character, therefore

***Resolved***, That the daily sessions of the association during each annual meeting, be divided into two parts—the first to terminate at an hour not later than 12 1-2 o'clock, P. M., of each day, and to be divoted as heretofore, to the general business of the association; the second, consisting of all the time which it is deemed advisable to remain in session each day, after 12 1-2 o'clock P. M., to take the character of a scientific section, and to be devoted exclusively to the discussion of questions relating to the science and art of medicine.

***Resolved***, That the association in its capacity of a scientific section, having no power to act on any subject except of a scientific character, may continue in session whenever thought advisable, a longer period than in its more general capacity.

***Resolved***, That the foregoing preamble and resolutions be referred to the committee of arrangements, with instructions to report on the same at the commencement of the next annual session.

The preamble and resolutions were agreed to.

Dr. A. J. Semmes, of Washington, District Columbia, offered the following, which was adopted:

***Resolved***, That a committee of three be appointed to report to the association at its next annual meeting what measure should be adopted to remedy the evils existing in the present methods of holding coroner's inquests, by incompetent persons, by which the lives and liberties of the innocent may be jeopardized, and the ends of justice frustrated.

Dr. Atlee offered the following which were adopted:

***Resolved***, That to secure efficient teaching in medical schools, where the prime object is to enforce practical precepts, a large degree of union and harmony must exist among the teachers, and confidence be reposed in them by their pupils.

*Resolved*, That any such unnatural union as the commingling of an exclusive system, such as homœopathy, with scientific medicine in a school, setting aside all questions of its untruthfulness, cannot fail by the destruction of union and confidence and the production of confusion and disorder, unsettling and distracting the minds of the learners, to so far impair the usefulness of teaching as to render every school adopting such a policy unworthy the support of the profession.

Dr. C. Stewart, of New York city, offered the following which were passed unanimously:

*Resolved*, That the thanks of the American medical association be, and they are hereby tendered to the committee of arrangements for the liberal and cordial reception extended to its members during the session.

*Resolved*, That a similar acknowledgement is eminently due, and also unanimously extended, to the officers, trustees, and managers of the several public and private institutions of the city and vicinity, which have been thrown open to the inspection of the members of the association, the visitation of which has been a source of mingled satisfaction, and the management of which manifests the faithful and zealous care of those to whose guardianship they have been entrusted.

The committee on nominations submitted a report, recommending that the resolution in reference to the registration of marriages, births and deaths be adopted, and that the following named members constitute the committee:

Dr. M. W. Wilson, Hartford, Conn., chairman.
" G S Palmer, of Gardiner, Me.
" Silas Cummings, of Fitzwilliams, N. H.
" G T Elliott, of Woodstock, Vt.
" Edward Jarvis, of Dorchester, Mass.
" Joseph Mauran, of Providence, R. I.
" John H Griscom, of New York.
" Henry Carpenter, of Lancaster, Pa.
" O H Taylor, of Camden, N. J.
" Lewis P Bush, of Wilmington, Del.
" A Snowden Piggot, of Baltimore, Md.
" D H Tucker, of Richmond, Va.
" —— Pittmann, of Tarb ro', N. C.
" Harry Lindsay, of Washington, D. C.
" John D Dawson, of Charleston, S. C.
" R D Arnold, of Savannah, Geo.
" A Lopez, of Mobile, Ala.
" James Jones, New Orleans, La.
" R C Foster, Nashville, Tenn.
" C J Blackburne, Covington, Ky.
" John Dawson, Columbus, O.

" Edward Murphy, New Harmony, Ind.
" A D Stebbins, Detroit, Mich.
" J V Z Blaney, Chicago, Ill.
" George D Wilbur, Mineral Point, Wis.
" William McPheeters, St Louis, Mo.
" J D Elbert, Reosaque, Iowa.
" John H Murphy, St Anthony Falls, Min.

The committee further reported in favor of the appointing of the following special committees:

Dr M M Latta, of Goshen, Indiana, "on whether there were any means by which the growth of the Fœtus in Utero may be controlled without injury to the mother or the child."

Dr Thomas Miller, of Washington, D. C., on Toxicology.

Dr E R Peasler, of Hanover, N H, on inflammation, its pathology and its relation to reparative process.

Dr D D Thompson, of Louisville, Ky, on the remedial effects of chloroform.

Dr Wm Clendennin, of Cincinnati, Ohio, on epidemic erysipelas.

Dr C G Comegys, of Cincinnati, Ohio, on the state of the urine in Turbercular disease.

The report was adopted.

Dr Clendennin, of Cincinnati, Ohio, offered the following:

*Resolved*, That no state or local society shall hereafter be entitled to representation in this association that has not adopted its code of ethics.

*Resoled*, That no state or local society that has intentionally violated or disregarded any article or clause in the code of ethics, shall longer be entitled to representation in this body.

Dr. Miltenberger, of Baltimore, moved the following additional resolutions:

*Whereas*, It has been brought to the notice of the American medical association that the state medical society of Ohio violated at their last meeting one of the articles of its code of ethics, Therefore,

*Resolved*, That the secretary of this association be directed to inform the officers of that society, that unless such action be rescinded, they cannot hereafter be represented in this association.

The resolutions were warmly advocated by several members, and finally adopted unanimously.

Several amendments to the constitution were offered, one of which provides that the travelling expenses of the secretary and treasurer shall be paid out of the general fund of the association. These amendments lay over until the next annnal meeting.

Dr. Corson read a report on the "influence of lead on the heart."

A number of resolutions making changes in the mode of imparting medical instruction were presented, and laid over for consideration at the next annual meeting.

A motion was made that the committee on publication have discretionary power in regard to the printing the report. The mover said that many of the members had not heard the reading of the report distinctly, and perhaps there may be some foolish remarks in it.

Dr. Corson replied that such a summary way of proceeding was not very courteous, so far as he was concerned. He had prepared his report with care, and he thought that the delegate might have given a more " delicate hint."

A motion was offered that it be referred to a special committee of three for examination, and if worthy of publication, to refer it to the proper committee for that purpose

The first motion was withdrawn, and the last was adopted.

The hour of recess now arrived, and a motion prevailed to dispense with the recess, and remain in session until final adjournment.

Dr. ——, of New York, moved that the time of holding the sessions of the association be changed from the first Tuesday in May to the second Tuesday. He said that this would better accommodate many of the delegates.

A delegate hoped it would prevail, to suit the members from New York.

Member of New York—There are seventy delegates there.

Member of Pennsylvania—You New Yorkers had better move on the first day of April, than on the 1st of May.—(Laughter.)

Member of New York—That's a customary rule and can't be avoided.

On motion, the whole subject was indefinitely postponed.

Dr Thomas, of Baltimore, explained briefly a very simple method of inhaling nitrate of silver for disease of the throat and chest, by converting it into powder. A small mixture for this purpose was exhibited.

Dr. Atlee called up a resolution offered at the last meeting of the Association, directing the especial attention of the committee on epidemics to the subject of contagiousness or uncontagiousness of cholera. The resolution was adopted.

A resolution was adopted tendering the thanks of the association to all railroad companies that have afforded members similar accommodations to those afforded by the Philadelphia and Wilmington and Baltimore railroad company. Adopted.

An amendment to the Constitution, offered at the last meeting, changing the time of electing the officers, was called up. The consideration of the amendment was lost.

A communication was received from Hon. R. T. Conrad, mayor, stating that an account of the reception of the delegates at Independence Hall, had been prepared for publication in pamphlet form, and would be ready for delivery to-morrow.

A resolution returning thanks to the Mayor, for this renewed expression of kindness, was passed.

Also, resolution of thanks to Dr. Horner, of the U. S. Navy, and Captain Marson of the U. S. Navy, for their invitations to visit the Navy Yard.

A delegate arose and said, that we have been so full of heart in passing resolutions of thanks by piecemeal, that perhaps we may have made some omissions. He would, therefore, move that the secretaries be instructed to fill up all omissions. Approved.

The resolution relating to the holding of coroner's inquests was referred to a special committee of three, to wit: Dr. Semmes of Washington, Dr. Hyle, of Wilmington, Dr. Condie, of Philadelphia.

At half past one o'clock, the association, on motion of Dr. Davis, adjourned *sine die.*

---

*American Medical Association.*

